# Supplementary material for: Peak visual gamma frequency is modified across the healthy menstrual cycle
Source: Hum Brain Mapp. 2018 Apr 17;39(8):3187–202. doi: 10.1002/hbm.24069 (PMC6055613; doi:10.1002/hbm.24069)
Supplement: Supplementary file 1 — Supporting Information [file HBM-39-3187-s001.docx]

**Supplementary material**


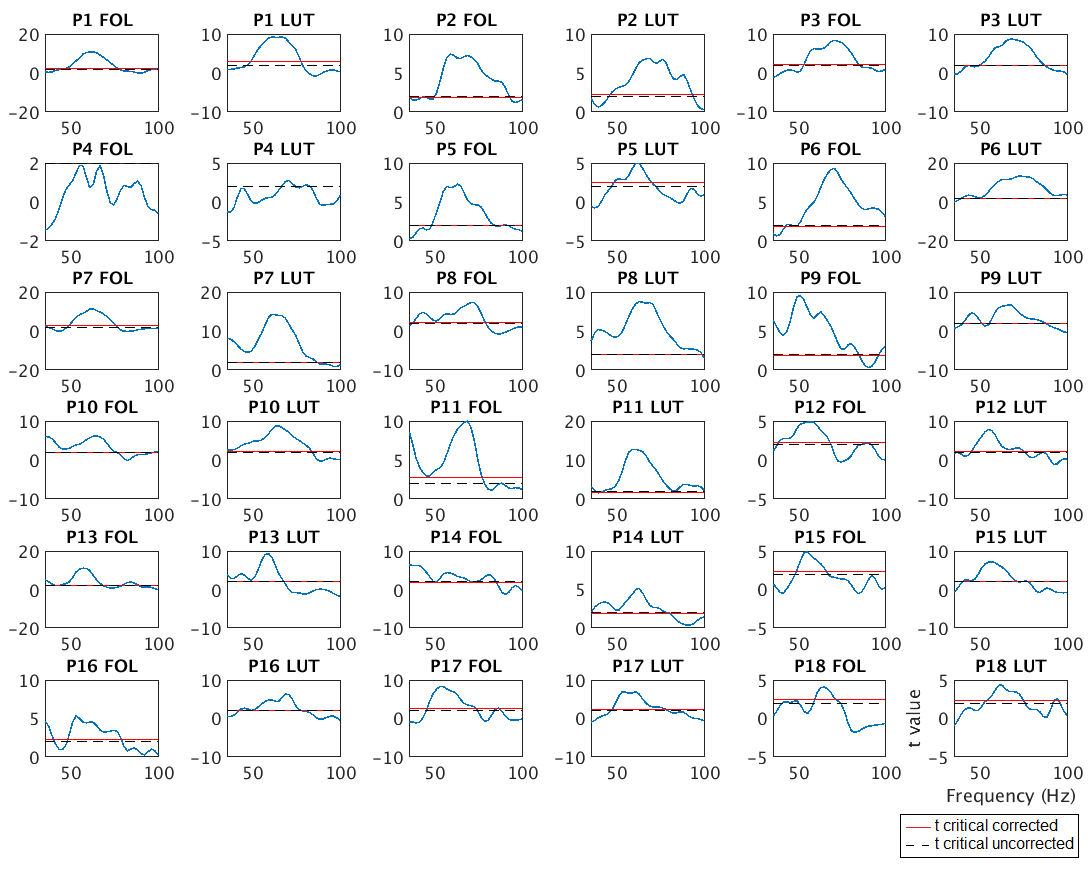


*Figure S1.* Moving gratings. Individual plots of signal enhancement showing the statistical t spectra for change in amplitude (stimulus induced gamma compared to baseline) as a function of frequency, computed across trials. The horizontal lines show t critical where *p* <0.05 uncorrected, or t critical corrected for multiple comparisons using FDR (for all *p*-values calculated between 35-90 Hz, that is, the same frequency window for the bootstrapped analyses). Where a t critical line cannot be seen, the value exceeded the data peak (P4 FOL).


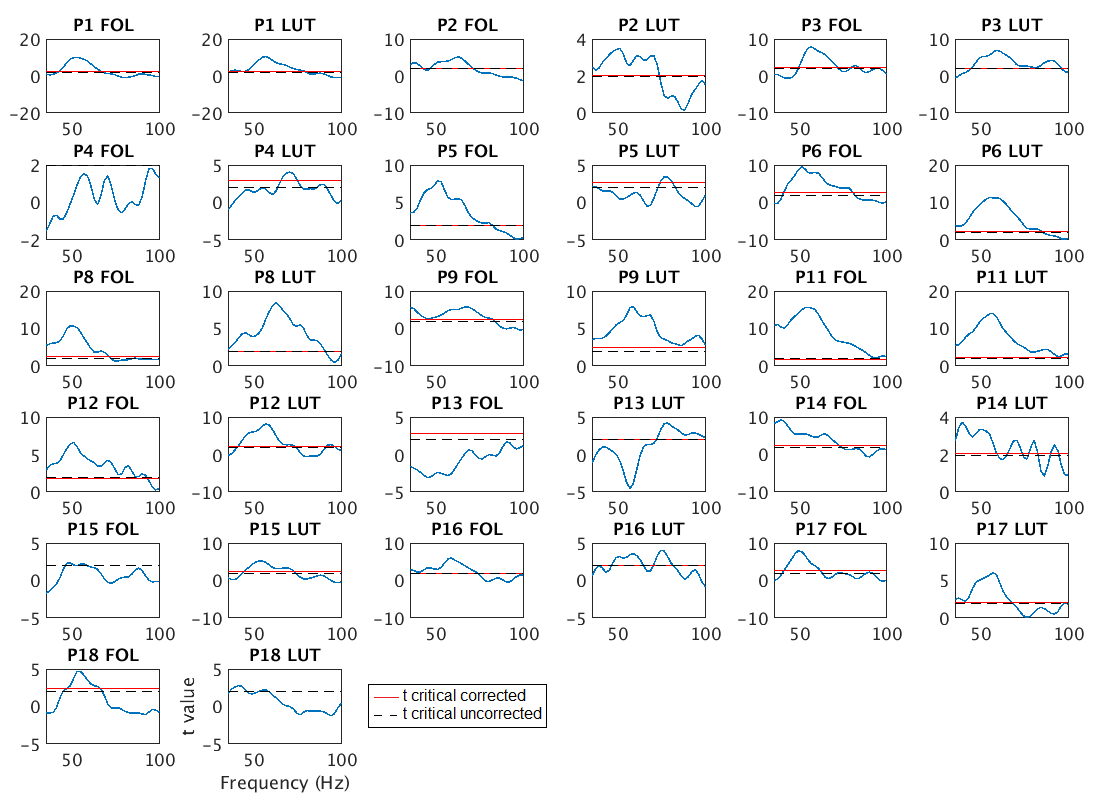


*Figure S2.* Static gratings. Individual plots of signal enhancement showing the statistical t spectra for change in amplitude (stimulus induced gamma compared to baseline) as a function of frequency, computed across trials. The horizontal lines show t critical where *p* <0.05 uncorrected, or t critical corrected for multiple comparisons using FDR (for all *p*-values calculated between 35-90 Hz, that is, the same frequency window for the bootstrapped analyses). Where a t critical line cannot be seen, the value exceeded the data peak (P4 FOL, P15 FOL, and P18 FOL). For static gratings it is evident that the datasets where t critical exceeds the data peak were also those marked as unreliable according to the bootstrapping quality control (QC) method. Due to the greater sensitivity of the QC method, additional datasets also fail to pass the threshold and were rejected from further analyses.
